# Using the Health Belief Model to explore why women decide for or against the removal of their ovaries to reduce their risk of developing cancer

**DOI:** 10.1186/s12905-018-0673-2

**Published:** 2018-11-14

**Authors:** Anne Herrmann, Alix Hall, Anthony Proietto

**Affiliations:** 1 Priority Research Centre for Health Behaviour, Health Behaviour Research Collaborative, University of Newcastle and Hunter Medical Research Institute, University Drive, Callaghan, 2308 Australia; 2 Cancer Services and Cancer Network, Hunter New England Local Health District, Newcastle, Australia

**Keywords:** Communication, Decision making, Oophorectomy, Patient-centred care, Qualitative research, Semi-structured interviews, Qualitative content analysis

## Abstract

**Background:**

Women at an increased risk of ovarian cancer often have to decide for or against the surgical removal of their healthy ovaries to reduce their cancer risk. This decision can be extremely difficult. Despite this, there is a lack of guidance on how to best support women in making this decision. Research that is guided by theoretical frameworks is needed to help inform clinical practice. We explored the decision-making process of women who are at an increased risk of developing ovarian cancer and had to decide for or against the removal of their ovaries.

**Methods:**

A qualitative study of 18 semi-structured interviews with women who have attended a cancer treatment centre or cancer counselling and information service in New South Wales, Australia. Data collection and analysis were informed by the Health Belief Model (HBM). Data was analysed using qualitative content analysis.

**Results:**

The paper describes women’s decision making with the help of the four constructs of the HBM: perceived susceptibility, perceived severity, perceived benefits, and perceived barriers. The more anxious and susceptible women felt about getting ovarian cancer, the more likely they were to have an oophorectomy. Women’s anxiety was often fuelled by witnessing family members suffer or die from cancer. Women considered a number of barriers and potential benefits to having the surgery but based their decision on “gut feeling” and experiential factors, rather than statistical risk assessment. Age, menopausal status and family commitments seemed to influence but not determine women’s decisions on oophorectomy. Women reported a lack of decision support and appreciated if their doctor explained their treatment choice, provided personalised information, involved their general practitioner in the decision-making process and offered a second consultation to follow-up on any questions women might have.

**Conclusions:**

These findings suggest that deciding on whether to have an oophorectomy is a highly personal decision which can be described with the help of the HBM. The results also highlight the need for tailored decision support which could help improve doctor-patient-communication and patient-centred care related to risk reducing surgery in women at an increased risk of ovarian cancer.

## Background

### Challenges of medical decision making

Cancer is the largest cause of death in Australia and worldwide, surpassing cardiovascular disease. On average one in two men and one in three women will be diagnosed with a form of cancer during their lifetime [1, 2]. Cancer incidence rates have been increasing over the last decades [3, 4]. Simultaneously, medical progress has resulted in a growing number of cancer prevention, screening and treatment options. Many patients have to make difficult decisions regarding the various options available to them [5–10]. More and more of these decisions involve options which show similar medical effectiveness but hold various side-effects and impacts that each patient may value differently. Such decisions are called “preference-sensitive” [11, 12]. Patients have to weigh-up the risks and benefits of the options available to them. The “best choice” cannot be pre-defined. It depends on patients’ preferences.

### Deciding for or against having an oophorectomy can be particularly difficult

Women at an increased risk of developing ovarian cancer may be offered a bilateral salpingo-oophorectomy, a surgical procedure to remove apparently normal ovaries and fallopian tubes in order to decrease their risk of developing cancer [13]. It is a particularly difficult decision to make, involving numerous risks and potential benefits which need to be taken into account [14]. The risks and benefits, and clinical recommendations can also vary depending on whether women carry certain gene mutations, their age and family history of cancer. Bilateral salpingo-oophorectomy has been shown to decrease women’s risk of developing ovarian cancer [15]. This is crucial for many patients as early ovarian cancer usually causes no or only very few symptoms. Ovarian cancer is often diagnosed at a stage where the cancer has spread beyond the ovaries [16]. The 5-year relative survival rate for Australian ovarian cancer patients is only 43% [1, 17]. As there is no proven screening method for ovarian cancer, having a bilateral salpingo-ophorectomy can significantly decrease patients’ anxiety and depression related to their perceived cancer risk [18]. Conversely, having an oophorectomy has been associated with short-term and long-term health risks, including surgical complications such as bleeding or infections, abrupt onset of menopausal symptoms such as hot flushes, and symptoms associated with menopause such as depression or anxiety [17]. Having the surgery may also cause an increased risk of cognitive impairment, osteoporosis and hip fracture [17]. Many patients experience a significant decrease in their sexual function and have to decide on whether to undergo hormone replacement therapy after having their ovaries surgically removed [19]. However, there is a lack of data on the effects of the long-term use of hormone replacement therapy and its psychological influences on women who underwent an oophorectomy [19].

### Research is needed to help women decide on this surgery

In order to adequately support women in deciding on whether or not to undergo the surgical removal of their ovaries, we need to understand why and how they make this decision. However, a recent review found a lack of research examining ovarian cancer treatment decision making from the perspective of the patient [20]. Uncertainty remains regarding the range and complexity of contextual factors that may impact on patients’ decisions [21–23]. Further, most research has been undertaken outside of Australia, and findings may not generalise given differences in healthcare delivery and social norms [23, 24]. Most studies failed to use a theoretical framework to guide research on decision making regarding oophorectomy. For example, a review of 43 studies on women’s decision making about risk-reducing strategies in the context of hereditary breast and ovarian cancer found that only two employed a theoretical framework to guide their research [23]. Employing a theoretical framework is likely to advance our understanding of women’s decisions about risk reducing strategies by helping to organise and integrate existing knowledge on preventive health behaviours. This can provide important guidance for research and clinical practice specific to the area of decision making on oophorectomy [23]. One model that may be particularly well suited to study women’s decisions on oophorectomy is the Health Belief Model (HBM). It was developed by a group of psychologists as a systematic method to explain and predict preventive health behaviour [25, 26]. The HBM is one of the most widely used theoretical frameworks for understanding health behaviour [27]. It is a psychosocial model that is designed to help understand health behaviours which prevent disease, or detect disease when a patient has little or no symptoms [28]. Unlike other models used to describe and predict health behaviour, such as the Theory of Planned Behaviour, the HBM focuses on intra-personal factors, including risk-related beliefs which influence individuals’ health-related decision making [29]. This was considered particularly important for this current study which aimed to explore women’s attitudes and beliefs regarding deciding on oophorectomy, with the aim to provide suggestions for clinical practice on how to better support this decision-making process.

Conducting qualitative research which is guided by theoretical frameworks can help provide valuable in-depth insights into patients’ perceptions of the decision-making process and thus enhance our understanding of existing quantitative data on patients’ views and experiences [30, 31]. Findings of qualitative research can further inform future studies by providing suggestions for how to design and implement decision support strategies [32]. Conducting qualitative research in this area will enable us to better help patients make difficult decisions and improve their outcomes.

## Methods

### Aims

To explore why women who are at an increased risk of developing ovarian cancer decide for or against the surgical removal of their ovaries.

### Design

A qualitative study of 18 semi-structured interviews.

### Setting and sample

Eighteen women who have attended a cancer treatment centre or cancer counselling and information service in New South Wales, Australia, took part in this study. Participants who were at an increased risk of developing ovarian cancer and had to make a decision regarding the surgical removal of their ovaries were included in this study. Women were defined as being at an increased risk of developing ovarian cancer if they have had at least one first degree relative diagnosed with ovarian cancer and/or carried a BRCA gene mutation [33]. A purposeful sampling frame was used to allow for recruitment of women who decided for removing their ovaries and those who decided against removing their ovaries. Data collection was stopped when data saturation was perceived to be reached and further data gathering was not considered to reveal additional findings to answer the research question [34, 35].

### Inclusion criteria

Eligible patients: (i) were determined by their treating clinician as being at an increased risk of developing ovarian cancer in the future (according to the criteria defined above); (ii) have decided for or against the surgical removal of their ovaries to reduce their cancer risk within the last two years (the outcome of this decision and when it was made was informed by the electronic patient management system described below); (iii) were aged 18 years or over; (iv) have attended a cancer treatment centre or cancer counselling and information service in New South Wales at least once in the past; (v) were determined by their treating clinician as physically and mentally capable of taking part in this study; and (vi) were English speaking.

### Recruitment

Potentially eligible patients were identified by their treating clinician through a state wide genetic services patient management system. It lists the names, decision for or against oophorectomy, BRCA gene status and contact details of women who have attended a cancer treatment centre or cancer counselling and information service in New South Wales. Eligible patients were mailed a study package by their treating clinician which included a study information sheet, a study consent form and a reply paid envelope. Patients who were willing to participate in the study returned the consent form to the research team by posting it in the provided reply paid envelope. Consenting patients were required to indicate their name, contact details and preferred contact time on the consent form.

### Data collection

Consenting patients were contacted by a member of the research team via telephone to arrange a time for a face-to-face interview, telephone interview or videoconferencing. Patients were given the choice of interview mode to reduce research related burden. The location of face-to-face interviews was determined according to what was most convenient for the patient. To further reduce research related burden, particularly for patients from remote areas, patients were given the option to conduct the interview via the free Avaya Scopia Mobile app. This software has been used by clinic staff for the transmission of clinical consultations by videoconference over the internet. Scopia offers patients the opportunity to receive some of their healthcare at or close to home through telehealth. It is completely secure, encrypted and confidential.

Before the interview commenced, patients were asked for permission to audio-record and transcribe the interview. They were informed that all data would be de-identified and thus remain confidential. Patients were further told that the interview would last approximately 30 min and that they could stop the interview or skip questions they did not feel comfortable answering any time. The interviewer then encouraged patients to tell their story about how they made the decision for or against the removal of their ovaries, in the way they prefer, with as little interruption as possible from the interviewer. This narrative approach helped elicit the range and interplay of potential reasons for or against having an oophorectomy [36, 37].

At the end of the narrative section, a set of semi-structured questions was used to explore particular issues further. These issues included reasons for why women decided for or against the surgery as well as factors which might have influenced their decision. The question guide was informed by the HBM which includes four constructs which were used to develop the questions patients were asked: perceived susceptibility, perceived severity, perceived benefits, and perceived barriers [38]. The questions were further informed by discussions amongst the research team which included experts in the areas of health behaviour, gynaecological oncology and qualitative research. Questions explored patients’:

*○ Perceived susceptibility to ovarian cancer* by asking patients how likely they thought it was that they will develop cancer in the future, how worried they were about developing cancer, how they felt about the lack of proven screening methods for ovarian cancer;

*○ Perceived severity of the situation* by asking patients how worried they were about the consequences of developing cancer (e.g. risk of dying, treatment side-effects, fear of outcomes, not being able to support their family while receiving treatment), whether they have had personal experiences with cancer themselves and/or in the family, whether this influenced their decision and if so, how;

*○ Perceived benefits of an oophorectomy* by asking patients whether they believed that the surgery would prevent cancer and if so, how much it would decrease their risk of developing cancer, whether they perceived it would decrease their worries related to developing cancer, whether they trusted the information they had received regarding their cancer risk and the surgery’s benefits, whether they felt the information was applicable to them and how it factored into their decision;

*○ Perceived barriers to an oophorectomy* by asking patients how they felt about potential complications of the operation (e.g. bleedings, infections), long-term effects of the surgery (e.g. abrupt onset of menopause), its impact on their sexuality, its impact on their ability to have children, its impact on “feeling like a woman”, its impact on social relationships (e.g. with their partner, family), financial issues (e.g. whether to take time of work, costs of the surgery), what impact the surgery may have on their family and other social obligations;

*○ Decision-making process* by asking patients what support they had in dealing with the consequences of the surgery and what else could have helped them make this decision, how much time they spent thinking about the surgery’s benefits and risks, and how long it took them to make the decision, who made the decision in the end and what happened after the decision was made.

Patients were also asked about the following sociodemographic and disease-related characteristics: highest level of education completed, occupation and marital status. To reduce research related burden for patients, further patient characteristics were sought with the help of their medical records, including date of birth, results of previous genetic testing and date of surgery (if applicable). Wherever possible standardised questions were used. All study materials were reviewed by the research team and pilot tested prior to finalisation with a group of health behaviour scientists and clinicians.

### Data analysis

All interviews were transcribed verbatim. Transcripts were double-checked for accuracy by a member of the research team (AH1). Text passages were read by members of the research team to familiarize themselves with the data and prepare the assignment of codes and categories (AH1 and AH2). A qualitative content analysis approach was chosen to identify and investigate key factors patients considered as influencing their decision regarding surgically removing their ovaries. Qualitative content analysis has been used frequently in nursing research, and is rapidly becoming more prominent in the medical and bioethics literature to systematically describe the meaning of qualitative data [39, 40]. This approach is particularly suited to multifaceted, sensitive phenomena, such as decision making on risk reducing surgery. Qualitative content analysis is recommended when there is no or only fragmented knowledge on the critical social process to be studied and meanings, intentions, consequences and context related to this process need to be investigated [30].

When conducting qualitative content analysis, text passages were coded following a systematic, interpretative act. Techniques used included a) summarising the data where the aim of the analysis was to reduce the material in such a way that the essential contents remain, b) explication of data where the aim of the analysis was to provide additional material on individual doubtful text components to increase understanding and interpreting particular passages of text, as well as c) structuring the data where the aim of the analysis was to filter out particular aspects of the material. These techniques were employed and results of the coding process were documented to help construct a coherent category system. This documentation contributed to the intersubjectivity of the procedure and would allow others to reconstruct or repeat the analysis [32].

The early stages of the coding process followed a conventional, inductive qualitative content analysis approach to minimise bias and ensure all relevant codes were captured. Initially, the transcripts were read line by line, and their content was examined, compared and categorized in order to apply a paraphrase or label (a “code”) that described what was interpreted in the passage as important. Codes were then grouped around the domains of the HBM to develop more abstract categories [30]. A category in this sense was a group of codes that shared a commonality [41]. If a code could not be linked to any of the domains, a separate category was developed to ensure all data was captured, regardless of whether it fitted in the existing model. This helped us validate and extend conceptually the underlying theoretical framework [42]. Based on the emerging categories, we generated threads of meaning across categories. Consequently, we analysed both latent and manifest content and chose each whole interview as unit of analysis [41]. Initial coding was conducted by one researcher (AH1). Conclusions drawn from the data were discussed amongst all three members of the research team (AH1, AH2, AP). In accordance with the principle of constant comparison, the robustness of the developed hypotheses was tested on different levels. As suggested by Przyborski and Wohlrab-Sahr, conclusions made by the research team were questioned on the basis of each single case as well as independently of individuals and thus beyond single cases [43]. Patient characteristics are presented using summary statistics. Chi-square tests were used to assess consent bias.

## Results

### Sample

Women were interviewed between March and November 2017. Eighty-six patients were invited to participate. Of these, 18 women (21%) consented to participate and were interviewed. Most interviews were conducted via telephone. Only one participant preferred to be interviewed face-to-face. Women had a mean age of 57 years, ranging from 22 to 81 years (SD = 15, see Table 1). Sixty seven percent of study participants had not been diagnosed with a gene mutation associated with an increased risk of developing ovarian cancer (*n* = 12). Of these, ten women (83%) had been tested for relevant gene mutations. Sixty one percent of women had previously been diagnosed with cancer (*n* = 11). Most of these women had been diagnosed with breast cancer (*n* = 9, 50%). Eleven participants underwent an oophorectomy, seven decided against the surgery. A mean of 23 days elapsed between study consent and interview (SD = 12). There were no statistical significant differences between consenters and non-consenters in terms of age and prevalence of relevant gene mutations (*p > 0.05*).

**Table 1:** Patients’ sociodemographic and disease-related characteristics

Characteristic	Patients (*n* = 18)
Age in years, mean (SD, range)	57 (15, 59)
18–39	17% (3)
40–59	39% (7)
60 or older	44% (8)
Menopausal status at the time of decision making	
Pre menopause	33% (6)
Currently undergoing menopause	6% (1)
Post menopause	61% (11)
Previous cancer diagnosis	
Yes	61% (11)
No	39% (7)
Marital status	
Married	44% (8)
De facto^a^	17% (3)
Divorced or separated	11% (2)
Single	28% (5)
Highest level of education	
Secondary school	50% (9)
Vocational	28% (5)
University	22% (4)
Gene mutations	
BRCA1	0 (0)
BRCA2	28% (5)
Other (e.g. Lynch Syndrome)	5.6% (1)
None	67% (12)
Oophorectomy	
Yes	61% (11)
No	39% (7)

^a^According to the Australian Department for Home Affairs a de facto partner relationship exists if all of the following applies: i) the partners are not legally married to each other; ii) they are committed to a shared life to the exclusion of all others; iii) their relationship is genuine and continuing; iv) they live together or do not live separately and apart on a permanent basis; v) they are not related by family [68]

### Perceived severity

Many women witnessed family members suffering and dying from breast or ovarian cancer. For most women who decided to have an oophorectomy, this was the single most important reason for getting their ovaries surgically removed. Several participants perceived undergoing cancer treatment to be worse than receiving a cancer diagnosis. They felt that having cancer treatment would have a strong impact on patients’ quality of life and lead to a lack of control over one’s health and wellbeing, even when a patient was in remission, due to the potential long-term treatment side-effects and fear of cancer recurrence.

*I don't have a problem with getting cancer, I have a problem with the treatment of cancer. From my own personal experience of seeing other people go through it, the treatment is worse than the disease. […] They're spending their whole life of not living their life, just thinking, oh, I've just to get to the next doctors visit and the next doctors visit. (Participant 7, 34 years, BRCA2 carrier, no oophorectomy)*

*Well, it's not easy for any woman but I guess maybe when you've been through something you make your decisions and you see people, you're losing people with ovarian and breast cancer, you do everything in your power to help yourself and help your family. (Participant 2, 68 years, BRCA2 carrier, oophorectomy)*

A number of women were concerned about the lack of symptoms of early-stage ovarian cancer and the fact that it is often detected at a late stage. Many women also worried about the non-specificity of symptoms of ovarian cancer. Most were aware of the low survival rates of ovarian cancer which highlighted to them the seriousness of the disease. This knowledge increased women’s fear of getting ovarian cancer and made them reflect on the potential benefits of having an oophorectomy. For many women, having their ovaries surgically removed seemed to be the only effective option of taking control over their perceived cancer risk.

*Basically if I felt bloated, if I was gaining weight or losing weight, or if I didn't feel right in my abdominal area then I should get professional medical help. I'm thinking, that's every other day I have these symptoms. It [=identifying relevant symptoms and seeking timely medical help based on self-assessment only] is not really realistic. (Participant 7, 34 years, BRCA2 carrier, no oophorectomy)*

### Perceived susceptibility

All women realised that they were at an increased risk of developing ovarian cancer. However, women’s perceived cancer risk often differed from their actual risk. Women who had cancer in the past or who had witnessed or heard of family members being diagnosed with a form of cancer often felt more susceptible to getting ovarian cancer than women without a personal or family history of cancer.

*Well it all started with my sister. She had cervical cancer. Yeah, so that’s how it all started. She had a gene test. We didn’t have the mutant gene, but I still felt that it would be less risk if I did have the op and to have them taken. I was particularly worried, my sister passed away at the beginning of this year, and to see her go through that wasn’t a good thing. That was certainly something that I never want to put myself through. So that was my main reason, I’d seen her go through it all, and so if I could just reduce that risk, I thought it would be a good start at least. (Participant 3, 68 years, BRCA2 carrier, oophorectomy)*

Also, women coped very differently with their perceived cancer risk. Many of the women who decided for the surgery did not carry a BRCA gene mutation. They were thus at a relatively low risk of developing ovarian cancer but could not cope with the anxiety related to their cancer risk. They felt that the cancer was “floating around” in their family (patient, 70 years) and thought that having the surgery would be the logical consequence to reduce their cancer risk. They considered the decision as a “no brainer” (patient, 57 years). In contrast, some of the women who had a BRCA2 gene mutation decided against the surgery, despite their relatively high risk of getting ovarian cancer. These women also felt susceptible to getting ovarian cancer but they felt less anxious and worried about their cancer risk. Women often trusted their “gut feeling” and based their decisions on experiences and beliefs, rather than statistical risk assessment. Although their decisions may seem to be emotive, women perceived their decisions to be perfectly logical and reasonable within the context of their experiences.

*I don't know whether some people would think I was crazy for making the decisions I’ve made, but I just feel – I don't know, my innate feeling – it's just a feeling that it's the right thing to do at the moment. (Participant 1, 60 years, no known gene mutations, no oophorectomy)*

### Perceived benefits

Reducing their risk of developing ovarian cancer and decreasing their worries related to their cancer risk were seen to be the most important benefits of having the surgery. Many women felt that taking away their worries would significantly improve their quality of life. This was although the data also revealed a lack of knowledge related to the potential benefits of undergoing an oophorectomy as some women felt that by having the surgery they would reduce their risk of ovarian cancer, whereas others thought it would prevent them from getting the cancer.

*But if I was living in fear and I was worried every day of my life that I may get cancer again then I probably would have had surgery. (Participant 1, 60 years, no known gene mutations, no oophorectomy)*

*I've had a lot of friends say that to me why would you want to have all that surgery if there's nothing wrong? I'm just like well, it's a ticking time bomb really. (Participant 7, 34 years, BRCA2 carrier, no oophorectomy)*

Women reported that a lot of the benefits of having an oophorectomy involved social factors. Many women decided for the surgery because they wanted to be able to be there for their children or grandchildren in the future. Some women also mentioned a need to be healthy in order to fulfil their family commitments, such as looking after their children or elderly parents and running a household. Some women said that they underwent the surgery to reduce the burden on others as the time and help they would need while recovering from the surgery would be much less than the time and help they would need following a cancer diagnosis and treatment.

*Probably one of my biggest fears, especially having a kid, that I wouldn’t be there for him. (Participant 9, 34 years, BRCA2 carrier, no oophorectomy)*

*As I said, I live on my own. I look after grandchildren during the school holidays. I’ve got my mum still alive, she’s 92. I’ve got to be fit and healthy. (Participant 4, 70 years, BRCA2 carrier, oophorectomy)*

### Perceived barriers

Women identified a number of barriers which they felt made it harder to opt for the surgery. For instance, several women feared the sudden onset of menopause and relating symptoms. They reflected on the potential intensity of symptoms, how long it would take them to physically, emotionally and mentally adjust to having early menopause and for how many years they may have to cope with menopausal symptoms.

*I don’t want to be this scarred cranky bitch, dry old woman in the body of a 40-year-old. And not just that, I've then got another 15 years on top of the normal woman who does that [= having menopause]. […] But I just want you to understand that I'm not a 67-year-old woman, I'm a 40-odd-year-old woman, so I've got to think of my long term. I'm fighting this to be here for a longer period of time, but I've got to think about these things so that I can manage in those years ahead. (Participant 14, 47 years, no known gene mutations, oophorectomy)*

Some women were concerned about the side-effects of hormone replacement therapy and that having this treatment could increase their risk of getting breast cancer. All women made their treatment decision based on what they thought would be the best long-term option for them.

*The risk factors of osteoporosis and that sort of thing that was relevant to me. I thought, oh, well maybe it's better if there's something else that we can do, as opposed to [having surgery and] then needing to take hormone replacement and all that sort of stuff, which puts you at higher risk of breast cancer once again anyway. (Participant 7, 34 years, BRCA2 carrier, oophorectomy)*

Some women were worried about potential surgical complications, such as bleeding or infections. Many women above the age of 60 felt more susceptible to such complications. Women tried to counteract these risks by choosing a clinic they found trustworthy, maintaining physical activity, planning sufficient recovery time and by seeking additional information through family, friends or support groups. Some women perceived the lack of an accurate risk assessment and the preventive (rather than curative) purpose of the procedure as a barrier to having the surgery. These women did not want to undergo an elective invasive procedure although they may never develop ovarian cancer. Others pointed out that there may be a chance that screening for ovarian cancer will be improved in the near future which could make the surgery redundant.

*There’s a difference between doing something for a purpose, but just in case seems to be subjecting your mind. Also when you get older anaesthetics aren't very good for your mental condition. It takes ages for you to get over anaesthetics, you know, as you get older as well. I didn't want my – how would I say? I didn't want my body violated, just in case. (Participant 5, 81 years, no known gene mutations, no oophorectomy)*

*When I had breast cancer, you just go, well you've just got to get the breast off. There's not a choice. It's got cancer, it's sick. It's an easy decision to make. I think I would be the same if I knew there was something wrong with my uterus or my ovaries or whatever. I'd say, yeah, absolutely take it. But when there's nothing wrong with them, as far as I know, it's harder for me to make that decision to have them out." (Participant 1, 60 years, no known gene mutations, no oopohrectomy)*

*And what if next year they find a test for it [=ovarian cancer]? Yes, that runs through your head. (Participant 11, 57 years, BRCA2 carrier, oophorectomy)*

Some women were concerned that the surgery might affect their femininity or their sexuality. They feared to have a decreased libido or may feel less like a woman. Women’s thoughts on this were also influenced by how supportive their partner was perceived to be in terms of having their ovaries surgically removed. However, for most women, impacts on their femininity or sexuality were of little or no concern as they felt that removing their ovaries would not change their appearance and thus be less obvious than other procedures, such as having a mastectomy. Some women even felt empowered by having an oophorectomy because they perceived that they made their own decision on their health and well-being. These women reported that deciding to get their ovaries removed made them realise that their femininity was linked to their attitudes and feelings, rather than to the presence of their ovaries.

*So for me that was a difficult decision because I was a woman that had gone into my 40s and reached my peak of … our children were now late teens, so my husband and I were getting more time to be intimate and all that sort of stuff. (Participant 14, 47 years, no known gene mutations, oophorectomy)*

*I mean you can't stand there at the café and say, oh, she's had a hysterectomy. I'd be more conscious if my breasts and things like that had to be removed. I mean, I haven't changed, my hair hasn't changed, my skin hasn’t changed. My husband hasn't said to me I'm getting crankier or not. (Participant 6, 55 years, no known gene mutations, oophorectomy)*

*I felt quite empowered because it just made me believe that all those things [=femininity and sexuality] are very much an inner thing and more of an emotional thing. (Participant 1, 60 years, no known gene mutations, no oophorectomy)*

Some women said that not being able to have children in the future was a barrier to undergoing the surgery. This was particularly relevant for younger women. Many of these women were advised by their treating doctor to have their children first and then undergo the surgery. Consequently, some women struggled with the pressure of having to finish their family plans in a timely manner in order to be able to have their ovaries removed. Some of the women who had a BRCA2 gene mutation decided against having further children because they were concerned that they may pass on the gene mutation.

*My next birthday I’ll be 34 and we’ve been trying to have a second child now for maybe seven or eight months and it still hasn’t happened yet. So in the back of my mind I feel like there is a bit of a deadline; I need to hurry up, but yeah. (Participant 9, 34 years, BRCA2 carrier, no oophorectomy)*

A number of women who their doctor considered to be too young to have surgery reported that they had not been offered a choice of whether to have their ovaries removed. They felt a lack of control over the decision-making process and wished that their doctor had explained the reason for this lack of choice more in detail. These women felt that this would have allowed them to feel more involved in and confident with their decision.

*I really kind of feel that I didn't really get a choice to tell you the truth because basically it was you're too young. (Participant 7, 34 years, BRCA2 carrier, no oophorectomy)*

Only a few women felt that financial barriers impacted on their decision. These women were single, on the pension or had to travel long distances to the clinic which meant that some of them had to take a considerable amount of time off work. However, for most women financial factors were not relevant as the costs for the surgery were covered by their health insurance and they lived close to the clinic where the surgery was done. Some women said that reducing their risk of having ovarian cancer would be invaluable which made the costs of having the surgery of little relevance to their decision.

*We had a big trip home, but then we stayed up there a couple of extra days so that it wouldn’t be as bad travelling home. You just try and plan. […] It is very, very difficult when you live in the country. (Participant 2, 68 years, BRCA2 carrier, oophorectomy)*

*Well, I'm on the pension, so I couldn't afford to pay for it. Yeah, because like I've been on the pension for a while and I just don't have that money to go ahead with it if it's going to cost me anything. (Participant 18, 74 years, no known gene mutations, no oophorectomy)*

*No it was all done free. I think the clinic covered the bills. I don't think money would have made a difference either. It was a long-term decision. (Participant 6, 55 years, no known gene mutations, oophorectomy)*

### Perceived self-efficacy

Women’s confidence in their ability to take action seemed to be an important factor for their decision on whether or not to have an oophorectomy. Women who decided for the surgery tended to feel the need to take control over their situation by having their ovaries surgically removed. They wished “to do everything they can” to decrease their cancer risk. These women felt that screening was invasive, strenuous and would not be effective in picking up ovarian cancer. They perceived the surgery to be the only option offering sufficient efficacy in risk reduction. In contrast, women who decided against the surgery felt that their ability to judge their health and wellbeing, to undergo regular screening and contact their specialist whenever symptoms arise or their personal circumstances change would give them sufficient peace of mind. This made having the surgery redundant.

*So I had to have checks all the time to see whether anything was going on. They’re really invasive tests, and they don’t guarantee, they’re not 100 per cent clear. So there was always that doubt. So in the end I was just sick of going through the tests that couldn’t really tell me conclusively anyway, so I decided to have everything removed. (Participant 11, 57 years, BRCA2 carrier, oophorectomy)*

*I thought, okay, well I'm feeling really good. I'm not seeing any history of ovarian cancer. I'll be keeping an eye on it. (Participant 1, 60 years, no known gene mutations, no oophorectomy)*

### Modifying factors and cues for action

A number of factors influenced women’s decisions on whether to have their ovaries surgically removed. For instance, age and menopausal status seemed to modify the decision-making process in many ways. All women felt that increasing age and being post-menopausal made it easier to decide for the surgery. This was linked to the perceived “uselessness” of ovaries once women had had children. However, there was no clear threshold indicating from what age women were more or less likely to undergo the surgery. For example, some women who were in their 30s decided for the surgery since they felt very anxious about their risk of developing ovarian or breast cancer. Some women were close to menopause or were post-menopausal but decided against the surgery because they feared that removing their ovaries could have a negative impact on their sexuality. Women’s medical history also influenced their decision, but as with age and menopausal status, this factor did not determine women’s decision. Some women who had been diagnosed with breast cancer in the past felt more alert and anxious about their cancer risk and decided for the surgery. Others wished not to have another invasive procedure since they had undergone breast cancer treatment and perceived this to be very burdensome. For example, some women had a mastectomy and feared that removing their ovaries would cause more scars on their body and further damage their feeling of being a woman.

*I've have my breasts taken […] but it has damaged my femininity, you know in my head space in what I see in the mirror it still affects me emotionally, and now you want to take away sort of my last part of my femininity. (Participant 14, 47 years, no known gene mutations, oophorectomy)*

Women reported a number of cues of action which promoted awareness and initiated the decision-making process. Some women reported that having family members recently undergoing genetic testing or passing away from cancer made them undergo genetic testing themselves. This often led to discussions with their doctor about whether or not to have the surgery. Other women reported that having their family plans completed would provide the cue to go ahead with the surgery.

*I mean it sort of put a bit more pressure on me, in that to finish having a family. It sort of put a bit of a timeline on it all. (Participant 9, 34 years, BRCA2 carrier, no oophorectomy)*

### The decision-making process

Most women reported that they made the final decision about whether to have an oophorectomy, after considering their healthcare providers’ opinions and involving their supportive others. The time women took to make the decision differed considerably, depending on women’s informational needs and preferences: While some women said they made their decision during or immediately following the initial consultation with their doctor, others took weeks or months to seek additional information and weigh-up the potential risks and benefits of having an oophorectomy. Consequently, not only the decision on whether to have an oophorectomy but also the decision-making process was highly personal and differed considerably depending on the needs and preferences of each individual patient.

*I think my husband and I more or less made it [=the decision] together. We’ve been together that long that we think the same anyway. (Participant 8, 62 years, no known gene mutations, oophorectomy)*

*It was just always in the back of my mind that that’s probably the better option to go. But I just needed to find more information about it before I actually made that decision because, yeah, it just affects, it does affect you, because all your hormones change and everything. So yeah, it’s a big change to get used to. (Participant 11, 57 years, BRCA2 carrier, oophorectomy)*

Many women could not remember exactly how high their risk of developing ovarian cancer was. Most women recalled being provided with this information but rather than basing their decision on numerical values, they focused on broad verbal categories (e.g. “high” or “low” risk) and experiential factors, such as their family history of cancer.

*We did have the general worry that we could get it [=ovarian cancer] because of so much cancer in the family. The doctor did give me some percentages, but I could not remember them now, on our chances of getting it. (Participant 8, 62 years, no known gene mutations, oophorectomy)*

A number of women reported asking other women who underwent the surgery about their experiences, and searching for additional information online. Some found online social networks helpful as they allowed them to read other patients’ experiences and ask questions. Thus, searching information online not only helped women overcome their perceived lack of knowledge but also provided some comforting by enabling them to share their experiences and thoughts with other women, whenever and wherever they wished to do so.

*There’s a few Facebook groups […] you sort of see more, you can read people’s circumstances or how their surgeries went. Because I don’t have family to talk really candidly about it. A lot of the stories on there are pretty open and honest and I find that helpful to read through them. […] You can post any questions you have on there and actually people that have gone through it or are thinking about going through it [answer], so that’s quite useful. (Participant 9, 34 years, BRCA2 carrier, no oophorectomy)*

*I wanted to be able to talk about it when I wanted to talk about it, when I could talk about it, and I didn't want to talk about it when I didn't want to talk about it. So I really think that that social component is really important. […] Rather than saying here's the medical reason why you need to do this, you know what I mean. (Participant 14, 47 years, no known gene mutations, oophorectomy)*

Many women felt a lack of support with choosing the right treatment option for them. Women perceived that compared with breast cancer, ovarian cancer received insufficient attention by researchers and policy makers, leading to a lack of public awareness and reliable information on ovarian cancer. Many women wished that they had had a better documentation of what had been discussed during the consultation with their doctor to help them “digest” and use the abundance and complexity of information they received. Women also indicated that this would have provided them with tailored and personalised information which they could not find by searching other information sources. Women appreciated having a follow-up consultation or the opportunity to call their specialist after their initial consultation.

*Everything seems to be the breast cancer. There’s not near as much with the other cancers and they really do need it. (Participant 15, 22 years, no known gene mutations, no oophorectomy)*

*Yeah, it was very difficult to find anything that really was representative of me. Like I could pull things out of different articles but there wasn't anything for the full-time student, single parent who didn't have a private health insurance. (Participant 7, 34 years, BRCA2 carrier, no oophorectomy)*

*If you can read the information and take it in yourself, then if you've got queries, ring back. Because sometimes you go to ring up and ask questions and they'll say something and it throws you and you forget the next question. So, I think if you can get your information and then ring them back, it's helpful. (Participant 2, 68 years, BRCA2 carrier, oophorectomy)*

Some women said it would have been helpful to see a short description of their specialists’ professional interests and experiences prior to the consultation. This would have allowed them to be reassured that their doctor has the expertise required to guide them through this preference-sensitive decision-making process. Many women found it very helpful to have their GP involved in the decision-making process in order to get a second opinion of someone they trust. This was also seen as an additional opportunity to ask questions women may have.

*Maybe a blurb or a CV on the doctor might, just a paragraph or two, might be handy. It might make people feel better if they know, I've done X amount of surgeries, I operate this often on blah, blah, blah, might put nervous people at rest. (Participant 6, 55 years, no known gene mutations, oophorectomy)*

*I have spoken about it with my own GP who is very, very thorough and she's supportive of everything I do. She researches it and she explained why it was that he had said that basically it wasn't an option for me at this point in time and I'm grateful. I know, a lot of people don't have access to a really good GP, and if I had any advice for anybody who was going through what I've essentially been through, it would be get yourself a good GP who knows what they're talking about or who is willing to investigate genetic issues. (Participant 7, 34 years, BRCA2 carrier, no oophorectomy)*

## Discussion

### Applicability and limitations of the Health Belief Model

Numerous studies have examined women’s decisions for or against having preventive surgery to reduce their cancer risk [23]. However, despite considerable research efforts in this area, there is a lack of theoretically guided studies that could help advance our understanding of patient decision making on oophorectomy [23]. Our study findings suggest that the HBM provides a structured approach to guide both data collection and analysis of research in this area, and helps describe women’s decision making on oophorectomy. As suggested by the HBM, women conducted an internal assessment of the benefits and barriers to undergoing the surgery, and then decided whether or not to act [29]. In line with research conducted in other geographical areas, women in our sample reflected on an array of factors before deciding on whether to undergo an oophorectomy, including potential complications of the surgery and long-term side-effects [23]. However, most women’s decisions were driven by their anxiety about developing ovarian cancer sometime in the future. Women’s decisions on whether or not to have an oophorectomy were thus often based on “gut feeling” and experiential factors, rather than statistical risk assessment. This is in line with previous studies describing the importance of experiential factors for individual decision making [44–46]. Our data suggest that witnessing a family member suffer or die from cancer can have a strong impact on women’s decision making who often feel more anxious about their own cancer risk and may thus be more likely to opt for the surgery. This also highlights the preference-sensitive nature of this decision and the need to provide appropriate support which can assist women in dealing with their familial experiences and choosing the option that is in line with their preferences.

Numerous studies have provided empirical evidence to support the dimensions of the HBM as important factors when explaining and predicting individuals’ health-related behaviours [27]. However, given its focus on attitudes and beliefs of individuals, the HBM does not involve all potential determinants that may dictate a person’s acceptance of a health behaviour [27]. Our study findings reflect this. For example, we also focused on the decision-making process to account for and explain data on decisional factors which were not captured by the original domains of the model. This approach also allowed us to make suggestions for clinical practice. It helped compensate for another limitation for the HBM which is more descriptive than explanatory, and does not suggest a strategy for changing health-related actions [27].

### Implication for clinical practice

Women appreciated being provided with a choice of whether to have an oophorectomy. Some clinicians may perceive that having their ovaries surgically removed is not an option for some patients, given that guidelines recommend oophorectomy to be considered for all women who are around the age of 40 and at an increased risk of ovarian cancer due to a confirmed BRCA1/2 gene mutation [47]. However, all women in our study indicated that they would still like to receive comprehensive information on the risks and potential benefits of the surgery, as well as details on why their doctor thinks they were not eligible for this procedure. This is in line with studies suggesting that the choice of treatment has an intrinsic value to patients, even if they decide to follow their doctor’s treatment recommendation [48]. Also, as suggested by previous research in this area, many women felt empowered by deciding on whether or not to get their ovaries removed. Not being offered a treatment choice made women perceive a lack of control over their situation and impacted negatively on their decisional confidence and satisfaction with the consultation with their doctor [49]. Consequently, it is important that clinicians try to guide women through this preference-sensitive decision-making process by explaining their choices, eliciting their preferences and tailoring care accordingly.

Many women reported a lack of information on the risks and benefits of having an oophorectomy. A documentation of what had been discussed during the consultation could help women recall and use the information provided to them. It would also allow women to access personalised and tailored information which they could not find elsewhere. Women appreciated if their doctor offered a second consultation to discuss their questions or concerns. This is in line with best practice guidelines on how to provide complex and potentially distressing information to patients [50, 51]. Having a follow-up consultation and being provided with additional information to consider in-between these consultations may help patients understand and weigh-up the information they received, seek further information, involve their support persons and increase their ability to participate in the decision-making process [52].

Given that it may not always be possible to cover all risks and potential benefits of all available treatment options during each consultation, tailored decision support, for example in the form of a decision aid, could supplement the above mentioned documentations of what has been discussed during the consultation and further support women with deciding on whether to have an oophorectomy. Patient decision aids present specific, evidence-based information on the healthcare options available to patients and aim to assist patients with clarifying and communicating the value they associate with each option [53]. They explicitly state the decision to be made and explain in detail the risks and potential benefits of the available options. Thus, decision aids help patients comprehend and weigh-up the risks and benefits of the options available to them and support patients in clarifying their preferences [54]. A decision aid on oophorectomy could include firsthand accounts of women who made this decision in the past since many women in our sample found it helpful to read about other women’s experiences. Decision aids on oophorectomy have been shown to improve a number of patient outcomes, such as increased knowledge of oophorectomy, decreased uncertainty related to their treatment options and decreased decisional conflict [55–57]. However, such strategies are still not commonly used in clinical practice which means that their benefits are unlikely to reach the intended patient populations [58]. This is in line with our findings indicating that many women perceived a lack of decision support when deciding on whether to have an oophorectomy. Further research on the influence of information experiences on women’s decision making, with particular focus on online information, may help provide valuable suggestions for how to design and test appropriate decision support strategies.

Also, women felt it would have been helpful to receive information on their doctor’s experiences and professional interests prior to the consultation. They thought that this would allow them to learn more about their clinician’s expertise, and help them choose the right healthcare provider for them. Previous studies have reported on the benefits of such information for doctor-patient communication and suggested that clinicians consider their online profile as an extension of their practice within reasonable limits [50, 59]. Additional clinician information provided online may facilitate healthcare decision making by increasing the trust patients place in their doctor [51, 52].

Women also indicated they appreciated the involvement of their general practitioners (GPs) in the decision-making process. This follows the principle of shared care which is the joint coordination and delivery of healthcare by a patient’s specialist and their GP [60]. Shared care has a number of potential benefits, including improved delivery and access to recommended healthcare [61], and improved coordination and continuity of care [62]. Involving women’s GPs in the decision-making process could help women feel more confident to ask any questions they may have, and get a second opinion from a healthcare provider who may be more familiar with their health and personal circumstances. Specialists could consider suggesting to patients that they see their GP prior to making the final decision.

### Limitations

Our results are not intended to be numerically representative. They rather provide in-depth insights into women’s decision-making process. We used a qualitative content analysis approach which was guided by the HBM. This may be criticized due to its potential to bias the analysis and thus the study findings [42]. However, to ensure a non-biased approach to coding which allows to identify and categorize all instances and dimensions of women’s decision-making process, we coded all transcripts inductively first and sought the HBM only to inform the later stages of the analysis. Consequently, we also developed codes and categories which did not fit into the model. Also, most interviews in our study were telephone interviews. Some authors may argue that this mode of data collection could be less valuable than face-to-face interviews. However, there is a lack of evidence on whether telephone interviews produce lower quality data [63, 64]. Also, most patients in our study preferred to be interviewed via telephone. They may feel more relaxed and able to disclose sensitive information when being interviewed on the telephone, and may find it easier to rearrange a telephone interview by calling back at a more convenient time, rather than having to rearrange a face-to-face interview [65].

Some women participated in the interview months after deciding on oophorectomy. This introduces the possibility of recall bias that could lead to inaccurate narratives. Also, most study participants had either a BRCA2 or no BRCA gene mutation. Women with a BRCA1 or other gene mutation have to consider different risks and potential benefits of having an oophorectomy and might thus have different experiences with deciding on oophorectomy [23]. Only few women mentioned CA125 screening as further option for monitoring their health. Future research should further explore the differences in women’s decision making related to specific gene mutations, as well as the role of CA125 screening in women’s decision-making process. Also, clinicians’ communication skills and styles may have influenced how women decided on whether to have an oophorectomy. For example, clinicians’ skills in communicating risks might have had an impact on patients’ understanding of their options [66, 67]. We did not record the consultations where the decision on oophorectomy was discussed. Thus, we do not know how clinicians’ communication skills and styles may have influenced patient decision making.

## Conclusions

This study identified and examined reasons for why women decided for or against the surgical removal of their healthy ovaries to reduce their risk of developing cancer. The results of this study suggest that deciding on whether to have an oophorectomy is a highly personal decision which can be described and explained with the help of the HBM. Many women trusted their “gut feeling” and based their decision on experiential factors, rather than statistical risk assessment. Our findings also emphasise the need for further decision support. This could be achieved by explaining women’s treatment choice, and providing them with both a documentation of what had been discussed during their consultation and a tailored decision aid. Women also appreciated the involvement of their GP in the decision-making process and found it helpful to be offered a follow-up conversation with a healthcare provider to address any concerns or answer any questions women may have. These strategies could help improve doctor-patient-communication and patient-centred care related to risk reducing surgery in women at an increased risk of ovarian cancer.

## Abbreviations

GP: General practitioners
HBM: Health Belief Model

## References

[1] Australian Institute of Health and Welfare & Australasian Association of Cancer Registries: Cancer in Australia: in brief 2014. Cancer *series no 91 Cat no CAN 89.* Canberra: AIHW; 2014.

[2] Ferlay J, Soerjomataram I, Dikshit R, Eser S, Mathers C, Rebelo M, Parkin DM, Forman D, Bray F (2015). Cancer incidence and mortality worldwide: sources, methods and major patterns in GLOBOCAN 2012. Int J Cancer.

[3] TW CD, Lu H (2010). Cancer in NSW: incidence and mortality report 2010.

[4] Kamangar F, Dores GM, Anderson WF (2006). Patterns of cancer incidence, mortality, and prevalence across five continents: defining priorities to reduce cancer disparities in different geographic regions of the world. J Clin Oncol.

[5] Brundage MD, Davidson JR, Mackillop WJ (1997). Trading treatment toxicity for survival in locally advanced non-small cell lung cancer. J Clin Oncol.

[6] Davis EL, Oh B, Butow PN, Mullan BA, Clarke S (2012). Cancer patient disclosure and patient-doctor communication of complementary and alternative medicine use: a systematic review. Oncologist.

[7] Goldhirsch A, Winer EP, Coates AS, Gelber RD, Piccart-Gebhart M, Thürlimann B, Senn H-J, Members P (2013). Personalizing the treatment of women with early breast cancer: highlights of the St Gallen international expert consensus on the primary therapy of early breast cancer 2013. Ann Oncol.

[8] Mauri D, Pavlidis N, Ioannidis JPA (2005). Neoadjuvant versus adjuvant systemic treatment in breast cancer: a meta-analysis. J Natl Cancer Inst.

[9] Okawara G, Rusthoven J, Newman T, Findlay B, Evans W (1997). Unresected stage III non-small-cell lung cancer. Cancer Prevent Control.

[10] Smith RA, Cokkinides V, Brooks D, Saslow D, Brawley OW (2010). Cancer screening in the United States, 2010: a review of current American Cancer Society guidelines and issues in cancer screening. CA Cancer J Clin.

[11] Brown R, Butow P, Wilson-Genderson M, Bernhard J, Ribi K, Juraskova I (2012). Meeting the decision-making preferences of patients with breast cancer in oncology consultations: impact on decision-related outcomes. J Clin Oncol.

[12] Politi MC, Lewis CL, Frosch DL (2013). Supporting shared decisions when clinical evidence is low. Med Care Res Rev.

[13] Das N, Kay VJ, Mahmood TA (2003). Current knowledge of risks and benefits of prophylactic oophorectomy at hysterectomy for benign disease in United Kingdom and Republic of Ireland. Eur J Obstet Gyn R B.

[14] Klitzman R, Chung W (2010). The process of deciding about prophylactic surgery for breast and ovarian cancer: patient questions, uncertainties, and communication. Am J Med Genet.

[15] Kauff ND, Satagopan JM, Robson ME, Scheuer L, Hensley M, Hudis CA, Ellis NA, Boyd J, Borgen PI, Barakat RR (2002). Risk-reducing salpingo-oophorectomy in women with a BRCA1 or BRCA2 mutation. N Engl J Med.

[16] Jacobs IJ, Menon U (2004). Progress and challenges in screening for early detection of ovarian cancer. Mol Cell Proteomics.

[17] Erekson EA, Martin DK, Ratner ES (2013). Oophorectomy: the debate between ovarian conservation and elective oophorectomy. Menopause.

[18] Hickey M, Ambekar M, Hammond I (2010). Should the ovaries be removed or retained at the time of hysterectomy for benign disease?. Hum Reprod Update.

[19] Madalinska JB, van Beurden M, Bleiker EM, Valdimarsdottir HB, Hollenstein J, Massuger LF, Gaarenstroom KN, Mourits MJ, Verheijen RH, van Dorst EB (2006). The impact of hormone replacement therapy on menopausal symptoms in younger high-risk women after prophylactic salpingo-oophorectomy. J Clin Oncol.

[20] Pozzar Rachel A., Berry Donna L. (2017). Patient-centered research priorities in ovarian cancer: A systematic review of potential determinants of guideline care. Gynecologic Oncology.

[21] Stacey D, Legare F, Col NF, Bennett CL, Barry MJ, Eden KB, Holmes-Rovner M, Llewellyn-Thomas H, Lyddiatt A, Thomson R (2014). Decision aids for people facing health treatment or screening decisions. Cochrane Database Syst Rev.

[22] Kinnersley P, Edwards A, Hood K, Cadbury N, Ryan R, Prout H, Owen D, MacBeth F, Butow P, Butler C. Interventions before consultations for helping patients address their information needs. Cochrane Database Syst Rev. 2007;(3):CD004565. 10.1002/14651858.CD004565.pub2 PMC9036848 17636767

[23] Howard AF, Balneaves LG, Bottorff JL (2009). Women’s decision making about risk-reducing strategies in the context of hereditary breast and ovarian cancer: a systematic review. J Genet Couns.

[24] Hallowell N, Baylock B, Heiniger L, Butow PN, Patel D, Meiser B, Saunders C, Price MA (2012). Looking different, feeling different: women's reactions to risk-reducing breast and ovarian surgery. Familial Cancer.

[25] Champion VL, Skinner CS: The Health Belief Model. Health behavior and Health Educ: Theory, research, and Practice Jossey-Bass 2008, 4:45–65.

[26] Hochbaum G, Rosenstock I, Kegels S: Health belief model. United States Public Health Service 1952.

[27] Janz Nancy K., Becker Marshall H. (1984). The Health Belief Model: A Decade Later. Health Education Quarterly.

[28] Stein JA, Fox SA, Murata PJ, Morisky DE (1992). Mammography usage and the health belief model. Health Educ Q.

[29] Green EC, Murphy E. Health belief model. In: The Wiley Blackwell encyclopedia of health, illness, behavior, and society: John Wiley & Sons, Ltd; 2014.

[30] Elo S, Kyngäs H (2008). The qualitative content analysis process. J Adv Nurs.

[31] O'Donnell S, Cranney A, Jacobsen MJ, Graham ID, O'Connor AM, Tugwell P (2006). Understanding and overcoming the barriers of implementing patient decision aids in clinical practice. J Eval Clin Pract.

[32] Mayring P (2000). Qualitative content analysis. Forum Qual Soc Res.

[33] National Breast and Ovarian Cancer Centre. Advice about familial apsepcts of breast cancer and ephelial ovarian cancer. A guide for health professionals. Sydney: Surry Hills; 2010.

[34] Coyne IT (1997). Sampling in qualitative research. Purposeful and theoretical sampling; merging or clear boundaries?. J Adv Nurs.

[35] Draucker CB, Martsolf DS, Ross R, Rusk TB (2007). Theoretical sampling and category development in grounded theory. Qual Health Res.

[36] Groleau D, Young A, Kirmayer LJ (2006). The McGill illness narrative interview (MINI): an interview schedule to elicit meanings and modes of reasoning related to illness experience. Transcult Psychiatry.

[37] Mishler EG. Research interviewing: context and narrative: Harvard University Press; 1991.

[38] Strecher VJ, Champion VL, Rosenstock IM: The health belief model and health behavior. Handbook of health behavior research 1: Personal and social determinants. New York: *Plenum Press* 1997:71–91.

[39] Forman Jane, Damschroder Laura (2007). Qualitative Content Analysis. Empirical Methods for Bioethics: A Primer.

[40] Schreier M. Qualitative content analysis. In: The Sage handbook of qualitative data analysis. Oxford: Sage; 2014. p. 170–83.

[41] Graneheim UH, Lundman B (2004). Qualitative content analysis in nursing research: concepts, procedures and measures to achieve trustworthiness. Nurse Educ Today.

[42] Hsieh H-F, Shannon SE (2005). Three approaches to qualitative content analysis. Qual Health Res.

[43] Przyborski A, Wohlrab-Sahr M. Qualitative Sozialforschung: Ein Arbeitsbuch: De Gruyter; 2014.

[44] Holmberg C, Waters EA, Whitehouse K, Daly M, McCaskill-Stevens W (2015). My lived experiences are more important than your probabilities:the role of individualized risk estimates for decision making about participation in the study of tamoxifen and raloxifene (STAR). Med Decis Mak.

[45] Hallowell N (2006). Varieties of suffering: living with the risk of ovarian cancer. Health Risk Soc.

[46] Hesse-Biber S, An C (2016). Genetic testing and post-testing decision making among brca-positive mutation women: a psychosocial approach. J Genet Couns.

[47] Cancer Australia. Recommendations for management of women at high risk of ovarian cancer. Surry Hills, NSW; 2011.

[48] Coulter A. (2010). Do patients want a choice and does it work?. BMJ.

[49] Hallowell N, Mackay J, Richards M, Gore M, Jacobs I (2004). High-risk premenopausal women's experiences of undergoing prophylactic oophorectomy: a descriptive study. Genet Test.

[50] Mostaghimi A, Crotty BH, Landon BE (2010). The availability and nature of physician information on the internet. J Gen Intern Med.

[51] Faber M, Bosch M, Wollersheim H, Leatherman S, Grol R (2009). Public reporting in health care: how do consumers use quality-of-care information? A systematic review. Med Care.

[52] Ball MJ, Lillis J (2001). E-health: transforming the physician/patient relationship. Int J Med Inform.

[53] International Patient Decision Aid Standards (IPDAS) Collaboration: What are Patient Decision Aids? URL: http://ipdas.ohri.ca/what.html. 10.1177/0272989X211035681 PMC8474333 34416841

[54] Lenz M, Buhse S, Kasper J, Kupfer R, Richter T, Mühlhauser I (2012). Decision aids for patients. Dtsch Arztebl Int.

[55] Hooker GW, Leventhal K-G, DeMarco T, Peshkin BN, Finch C, Wahl E, Joines JR, Brown K, Valdimarsdottir H, Schwartz MD (2011). Longitudinal changes in patient distress following interactive decision aid use among BRCA1/2 carriers:a randomized trial. Med Decis Mak.

[56] Metcalfe KA, Poll A, O’Connor A, Gershman S, Armel S, Finch A, Demsky R, Rosen B, Narod SA (2007). Development and testing of a decision aid for breast cancer prevention for women with a BRCA1 or BRCA2 mutation. Clin Genet.

[57] Tiller K, Meiser B, Gaff C, Kirk J, Dudding T, Phillips K-A, Friedlander M, Tucker K (2006). A randomized controlled trial of a decision aid for women at increased risk of ovarian cancer. Med Decis Mak.

[58] Elwyn G, Scholl I, Tietbohl C, Mann M, Edwards AG, Clay C, Legare F, van der Weijden T, Lewis CL, Wexler RM (2013). “Many miles to go ...”: a systematic review of the implementation of patient decision support interventions into routine clinical practice. BMC Med Inf Decis Mak.

[59] Edgman-Levitan S, Cleary PD (1996). What information do consumers want and need?. Health Aff.

[60] Smith SM, Allwright S, O'Dowd T (2007). Effectiveness of shared care across the interface between primary and specialty care in chronic disease management. Cochrane Database Syst Rev.

[61] Earle CC, Neville BA (2004). Under use of necessary care among cancer survivors. Cancer.

[62] Aubin M, Giguere A, Martin M, Verreault R, Fitch MI, Kazanjian A, Carmichael PH (2012). Interventions to improve continuity of care in the follow-up of patients with cancer. Cochrane Database Syst Rev.

[63] Holt A (2010). Using the telephone for narrative interviewing: a research note. Qual Res.

[64] Irvine A (2011). Duration, dominance and depth in telephone and face-to-face interviews: a comparative exploration. Int J Qual Methods.

[65] Novick G (2008). Is there a bias against telephone interviews in qualitative research?. Res Nurs Health.

[66] Jansen J, van Weert JC, Wijngaards-de Meij L, van Dulmen S, Heeren TJ, Bensing JM (2010). The role of companions in aiding older cancer patients to recall medical information. Psychooncology.

[67] Wills CE, Holmes-Rovner M (2003). Patient comprehension of information for shared treatment decision making: state of the art and future directions. Patient Educ Couns.

[68] Australian Department of Home Affairs: What a de facto relationship is. URL: https://www.homeaffairs.gov.au/visas/supporting/Pages/partner/what-de-facto-relationship-is.aspx.

